# ADVANCING AGE INCLUSIVITY IN HIGHER EDUCATION: INTERGENERATIONAL EXCHANGE AS A PATHWAY TO CAREER READINESS

**DOI:** 10.1093/geroni/igad104.0469

**Published:** 2023-12-21

**Authors:** Joann Montepare, Kimberly Farah, Lisa Borrero

## Abstract

Higher education is becoming more age-inclusive on several fronts and age-friendly teaching and learning strategies are being used in new and novel ways to support a variety of students’ educational outcomes. In this AFU-ILRCE collaborative symposium, presenters will discuss ways that intergenerational exchange in the classroom and beyond can help to prepare students for entry into the workforce. To begin, Montepare, Farah, and Frazier will give an overview of the Age-Friendly University (AFU) initiative and discuss how older adults can serve as teaching allies in career-focused classroom activities such as serving as job recruiters in mock interviews. Graf and Terhune will share reflections from focus group data on intergenerational exchanges in the workplace conducted with working and recently retired adults 60+ years of age. They will highlight the utility of such perspectives for informing career training programs for students regardless of major or career path. Mastel-Smith, Kimzey, and Garner will describe Dementia Bootcamp, an online program for high school students that utilized didactic information, experiential activities, presentations by people with dementia, and volunteering at a dementia day program. Focus group findings and how the program has been integrated into a health sciences curriculum also will be presented. Ghazi-Saidi & McKelvey will discuss an innovative experiential learning activity in two core courses of a Speech Language Pathology (SLP) graduate program that prepares future SLPs to use telepractice to provide cognitive health interventions to community older adults. Lisa Borrero, chair of AGHE’s Education Resource Development Workgroup (ERDW) will serve as discussant. This is a collaborative symposium between the Age-Friendly University (AFU) Network and Intergenerational Learning, Research, and Community Engagement Interest Groups.

